# Catheter ablation of atrial fibrillation in a patient with interruption of the inferior vena cava complicated with persistent left superior vena cava

**DOI:** 10.1002/joa3.13018

**Published:** 2024-03-05

**Authors:** Li Shu, Yi Lu, Shenghui Ma, Chunhui Liu, Zhejun Cai

**Affiliations:** ^1^ Department of Cardiology Second Affiliated Hospital of Zhejiang University School of Medicine Hangzhou Zhejiang China

**Keywords:** atrial fibrillation, interruption of the inferior vena cava, persistent left superior vena cava, radiofrequency catheter ablation

## Abstract

A 55‐year‐old woman of I‐IVC complicated with PLSVC underwent catheter ablation for atrial fibrillation through right jugular vein access. TSP was achieved by electrocautery and the J‐tip guidewire with the help of deflectable sheath and ICE. After PVI, the CS‐PLSVC and LA‐PLSVC connections were ablated within PLSVC.

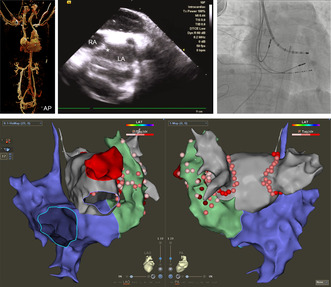

Interruption of the inferior vena cava (I‐IVC) with azygos or hemiazygos continuation at the level of the liver is a rare vascular variant with a reported prevalence rate of about 0.6%–2%.^1^ There were few reports of catheter ablation for atrial fibrillation in these patients with atrial fibrillation (AF). According to the reports, superior vena cava, hepatic vein, or aortic approach can be used.^2^

Persistent left superior vena cava (PLSVC) is the most common anomaly of the venous system, and the incidence will increase to 10% in patients with congenital heart malformation.^3^ It has been widely accepted that PLSVC could be the arrhythmogenic source of atrial fibrillation, suggesting the importance of PLSVC as an ablation site.^4^ However, when complicated with I‐IVC, the catheter ablation becomes extremely challenging. Here, we present a successful catheter ablation of AF in a patient complicated by I‐IVC and PLSVC under the guidance of intracardiac echocardiography (ICE).

A 55‐year‐old woman with symptomatic persistent AF was scheduled for catheter ablation 6 years ago. The patient experienced occasional chest tightness and palpitation. An electrocardiogram (ECG) showed paroxysmal AF with a rapid ventricular rate. Intra‐operative angiography before catheter ablation showed I‐IVC and PLSVC. Therefore, the procedure was aborted. She was prescripted with amiodarone 200 mg/day and rivaroxaban 15 mg/day. The patient underwent permanent pacemaker implantation due to sinus bradycardia 5 years ago. However, in the past year, the patient's chest discomfort recurred, and pacemaker programming data revealed persistent AF in the patient.

The baseline rhythm was AF with ventricular pacing rhythm. The echocardiogram showed PLSVC with enlarged coronary sinus ostium. CT venography confirmed that the patient had separate SVC and PLSVC, complicated by interrupted IVC. The left and right common iliac veins merged into the azygos vein instead of IVC and then drained into the left subclavian vein, which finally entered the right atrium through the PLSVC (Figure 1A). The hepatic veins drain directly into the low RA. In addition, situs inversus partialis with transposition of abdominal organs was indicated.

**FIGURE 1:**
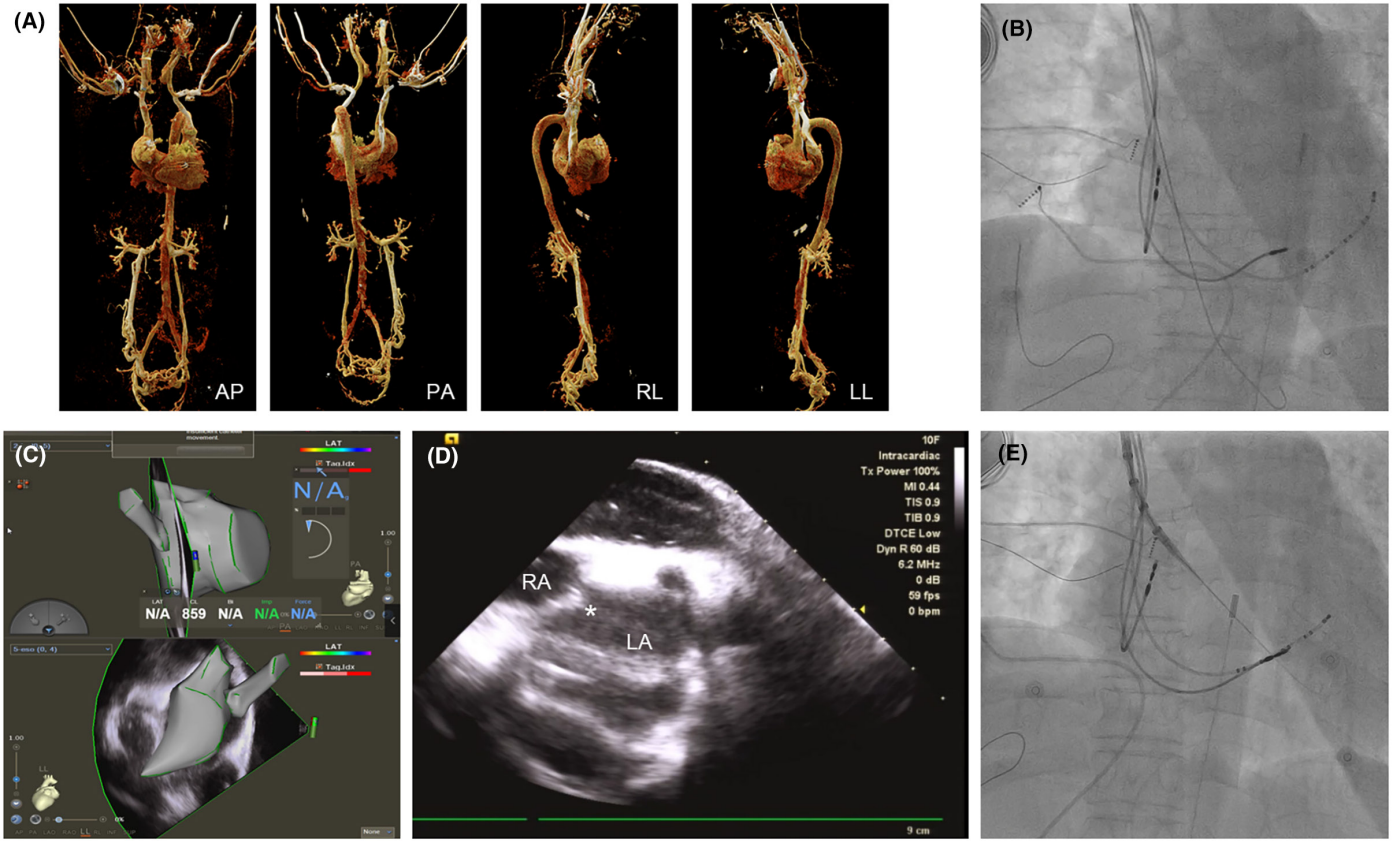
Transseptal puncture in this case. (A) CT venography of the patient with interruption of the inferior vena cava (I‐IVC) complicated with persistent left superior vena cava (PLSVC) in different projections. The patient has separated SVC and PLSVC. The azygos vein instead of IVC drained into the left subclavian vein and finally entered the right atrium through the PLSVC. (B) The decapolar catheter was placed into coronary sinus through the right jugular vein. The 10F intracardiac echocardiography (ICE) catheter was placed at the level of the LA in the azygos vein through the left femoral vein. (C) ICE modeling of the left atrium. (D) Visualization of transseptal puncture (TSP) by ICE. The asterisk indicated the sight of tenting by VIZIGO sheath. (E) The X‐ray image after TSP. The J‐tip guidewire was in the left ventricle.

After transesophageal echocardiography (TEE) with screened‐out thrombus in the left atrium or left atrial appendage, the patient was subjected to catheter ablation of AF with written informed consent obtained. The procedure was conducted under local anesthesia and conscious sedation.

The ICE catheter (10 Fr, SoundStar; Biosense Webster, Irvine, CA) was placed at the level of the LA in the azygos vein through the left femoral vein (Figure 1B). The anatomic map of LA was then created using the 3D navigation system (CARTO; Biosense Webster, Irvine, CA) (Figure 1C). A decapolar catheter (Biosense Webster, Irvine, CA) was placed into the coronary sinus (CS) through the right jugular vein (Figure 1B). Because of the pacemaker leads, the ICE was not placed in the RA. Next, a large curve VIZIGO sheath (Biosense Webster, Irvine, CA) was placed into RA through the right jugular vein. The VIZIGO sheath was curved and the tip was pointed to LA in the higher oval fossa under the guidance of ICE with the J‐tip guidewire (Biosense Webster, Irvine, CA) inside the sheath (Figure 1D). RF power (20 W for 2 s) was manually applied to the proximal end of the guidewire using an electrocautery pen (Shuyou, Zhejiang, China) to accomplish transseptal puncture (TSP) as described previously^5^ (Figure 1E).

Next, a multipolar electrode catheter (PentaRay; Biosense Webster, Irvine, CA) was inserted into LA along the VIZIGO sheath for further advanced LA modeling (Figure 2A, B). The PentaRay was replaced by an ablation catheter (SmartTouch Surround Flow, STSF; Biosense Webster, Irvine, CA) afterward, and bilateral extensive PV isolation was completed (power 50 W, contact force 5–20 g, and ablation index 400–550) followed by electrical cardioversion to restore sinus rhythm (Figure 2C–G). Due to the short course of persistent AF, no further linear ablation was applied. Because the PLSVC is a possible trigger of AF, it was further electrically isolated. The anatomic model of PLSVC was created by PentaRay (Figure 3A). The STSF catheter was then inserted to isolate PLSVC with LA and CS within PLSVC. The CS‐PLSVC connection was first isolated at the level of lower LSPV. After that, LAA pacing was performed, and the LA‐PLSVC connection was ablated within PLSVC. The target AI was 350–400. Finally, the STSF catheter was placed at PSLVC adjacent to the LA side to confirm that PLSVC‐LA was electrically isolated (Figure 3B,C).

**FIGURE 2:**
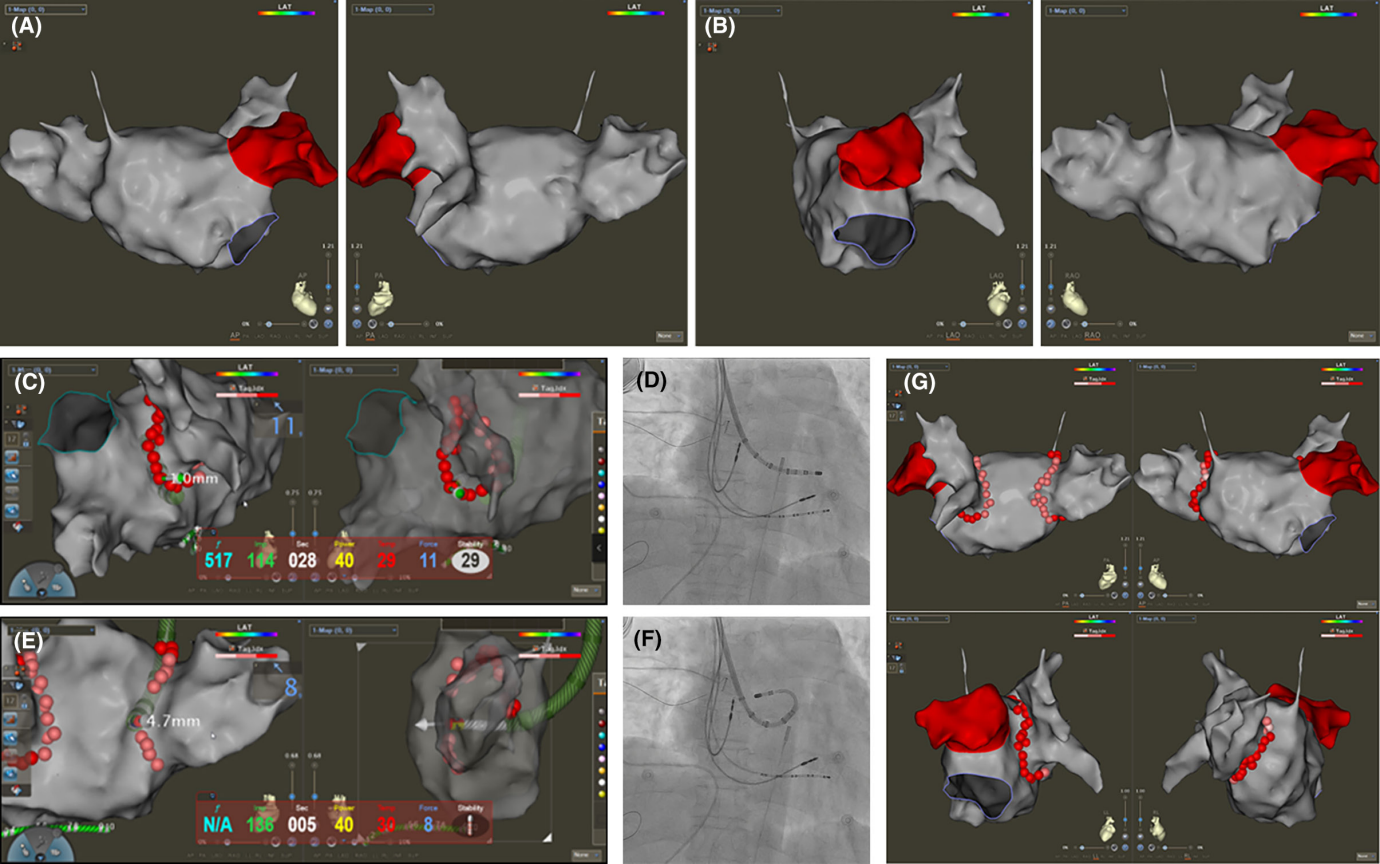
Pulmonary vein isolation in this case. (A, B) Advanced anatomic modeling of the left atrium by PentaRay catheter. (C) Isolation of left superior and inferior pulmonary veins. (D) The X‐ray image shows the position of ablation catheter during isolation of the left inferior pulmonary vein. (E) Isolation of right superior and inferior pulmonary veins. (F) The X‐ray image shows the the position of ablation catheter during isolation of the right superior pulmonary vein. (G) Completion of pulmonary vein isolation.

**FIGURE 3:**
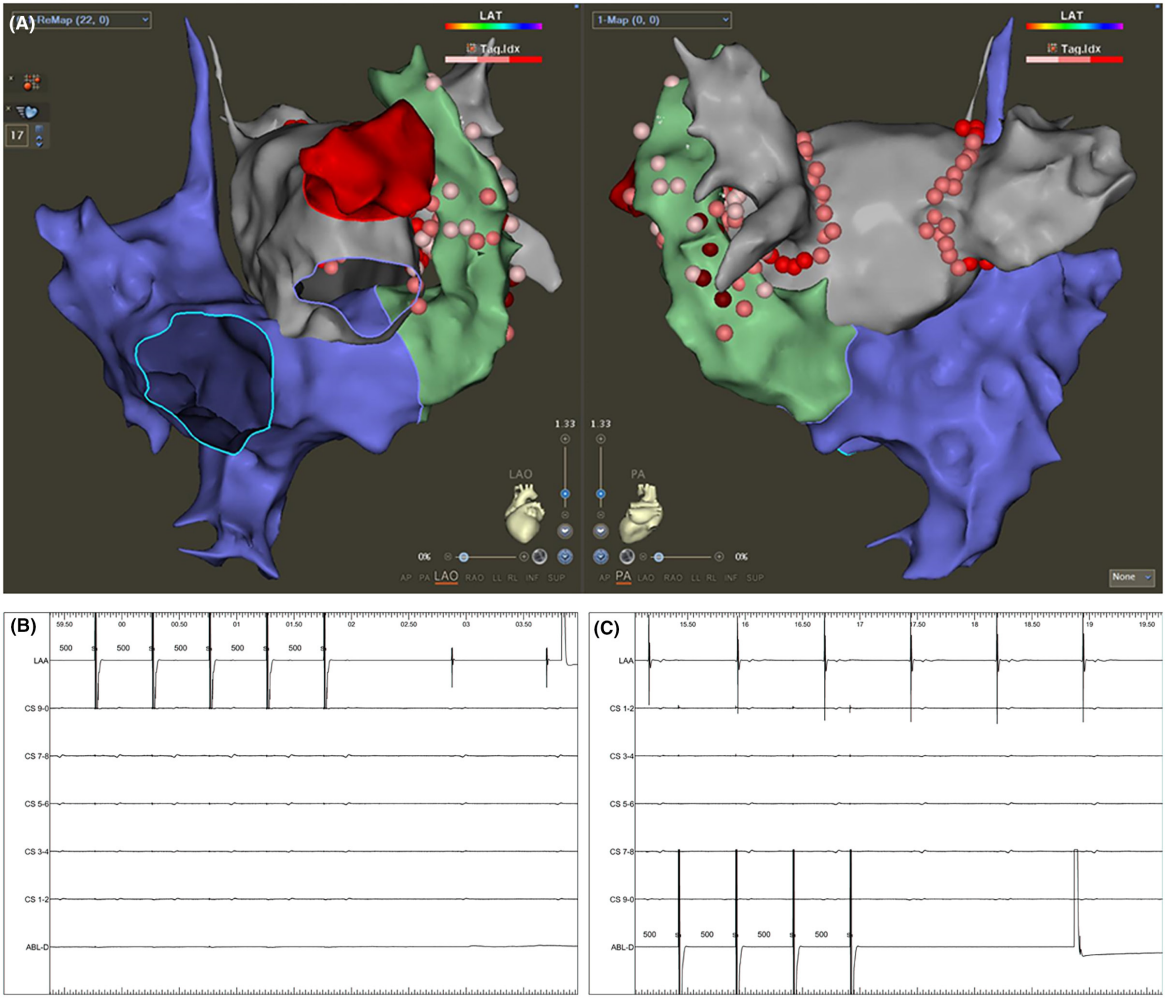
Isolation of persistent left superior vena cava in this case. (A) Anatomic modeling of persistent left superior vena cava (PLSVC) by PentaRay catheter, and the final ablation points for isolation of left atrium (LA)‐PLSVC and coronary sinus (CS)‐PLSVC connection. The PentaRay catheter was placed in left atrial appendix, Decapolar catheter was placed inside mid‐distal PLSVC, and the ablation catheter was placed at PSLVC adjacent to the LA side. Pacing from LAA (B) and from mid‐distal PLSVC (C) confirmed the elimination of local potential and loss of CS‐PLSVC and LA‐PLSVC connection.

Finally, an injection of isoproterenol and ATP was applied to confirm PVI and exclude other ectopic triggers. A set of atrial burst pacing for 300 ms per 8 beats, and the time was reduced by 20 ms at every time point until 160 ms was performed, and AF could not be induced.

The total procedure time was 109 min. At 6‐month follow‐up, the patient maintained sinus rhythm and was asymptomatic.

This case highlights the use of ICE and electrified guidewire‐assisted TSP in complicated AF cases. In a previous report, a curved BRK needle was applied for TSP in I‐IVC. However, when combined with PLSVC, and TSP access becomes much more challenging due to dilated coronary sinus. The use of ICE makes it possible to perform TSP in such complicated cases under conscious sedation and avoid using TEE. ICE placed in azygos vein also could provide a clear view for TSP. The electrified guidewire‐assissted TSP has been proven to be an alternative TSP approach. Compared with a general fixed‐angle sheath, using of deflectable sheath can help to adjust the TSP site more precisely and make it easier and safer to get access to the TSP site guided by ICE. PLSVC plays an important role in the formation and maintenance of AF. Studies have proven that ablation of PLSVC can reduce the recurrence rate of AF.^4^ The current case also provides evidence that PLSVC isolation can be accomplished via the superior vena cava approach.

## FUNDING INFORMATION

This work was supported by funding from the National Natural Science Foundation of China (nos. 81970396 and 82270497 to Zhejun Cai) and the Zhejiang Provincial Natural Science Foundation for Distinguished Young Scholars (no. LR20H020002 to Zhejun Cai).

## CONFLICT OF INTEREST STATEMENT

The authors declare no conflict of interest.

## ETHICS STATEMENT

The study was approved by the Ethics Committee of the Second‐Affiliated Hospital, Zhejiang University School of Medicine.

## PATIENT CONSENT STATEMENT

The patient signed the informed consent.

## CLINICAL TRIAL REGISTRATION

N/A.

## Data Availability

The data that support the findings of this study are available from the corresponding author upon reasonable request.
